# Low seroprevalence of hepatitis B virus and absence of hepatitis C virus among patients attending dental clinics in Mogadishu, Somalia

**DOI:** 10.1186/s12876-026-04846-x

**Published:** 2026-04-20

**Authors:** Omar Osman Mohamed, Bashir Abdirahman Guled, Abdirahman Hussein Elmi

**Affiliations:** Department of Medical Laboratory Sciences, Faculty of Medicine and Health Sciences, Jamhuriya University of Science and Technology, Mogadishu, Somalia

**Keywords:** Hepatitis B, Hepatitis C, Seroprevalence, Dental clinic, Somalia, Public health

## Abstract

**Background:**

Hepatitis B virus (HBV) and hepatitis C virus (HCV) represent major global health challenges, especially in areas where healthcare resources are restricted. This study estimated the seroprevalence of HBV and HCV among patients visiting a selected dental clinic in Mogadishu, Somalia.

**Methods:**

This cross-sectional study was carried out in Alfurad Dental Clinic between February 2023 and March 2024. A sample size of 410 participants was calculated using Cochran’s formula. Data was collected using structured interviews along with serological testing for hepatitis B surface antigen (HBsAg) and hepatitis C antibody (anti-HCV). Statistical analyses were conducted using SPSS (version 27.0), with a significance level set at *p* < 0.05.

**Results:**

Among the 410 participants, the seroprevalence of HBV was 2.0% (95% CI: 0.9% to 3.6%), with no cases of HCV reported. The majority of participants were female (73.7%). The highest prevalence of HBV was observed in the 60–69 years old age group, and 45.9% of participants had non-formal education. The proportion of individuals vaccinated against hepatitis B was low, at 3.2%, and none of them were found to be infected. No statistically significant associations were identified between HBV prevalence and the following demographic variables: age, gender, marital status, and educational level, or medical history (*p* > 0.05).

**Conclusions:**

The present study reveals a low prevalence of HBV among dental patients in Mogadishu, with a seroprevalence of 2.0%. There were no reported cases of HCV. The results highlight the importance of improved public health strategies that prioritize screening and vaccination to reduce the risk of hepatitis transmission within this particular population. Further investigation is crucial to track developments and guide specific actions.

## Introduction

Hepatitis B virus (HBV) and hepatitis C virus (HCV) persist as significant health problems [1, 2], impacting roughly 325 million individuals worldwide and accounting for 96% of all hepatitis-related mortality globally, and the majority remain undetected or untreated [3, 4]. HBV and HCV can progress to chronic disease, which leads to liver cirrhosis and hepatocellular carcinoma (HCC) [5–9]. Both have similar transmission pathways, and co-infection with both viruses is prevalent [10, 11]. They can be transmitted via vertical (mother-to-child) transmission, sexual contact, sharing of infected personal items, or through healthcare procedures using infected tools or blood transfusions [12–14].

In 2016, the World Health Organization (WHO) declared a strategy to eradicate viral hepatitis as a public health concern by 2030 [15]. This objective could be difficult, particularly in resource-constrained regions of the globe. Challenges to eradication efforts in resource-constrained regions encompass inadequate diagnostic facilities and insufficient public awareness [3].

There is a distinct geographical variation in both HBV and HCV prevalence and incidence due to a lack of proper health facilities, poor economic status, and limited public awareness about the transmission of major communicable diseases [8, 16].

Due to their shared transmission modes, HBV and HCV infections present significant problems for dental practitioners [17]. Dentists face a higher chance of infection from patients’ saliva and blood [17, 18]. Before dental treatment, a comprehensive health history must be acquired to identify individuals with hepatitis [19]. Serological tests or molecular diagnostics can diagnose HBV and HCV [20].

The seroprevalence of HBV and HCV infections in the Somali population needs to be better estimated, especially among individuals seeking health care services. Dental care facilities represent an essential access point for several individuals seeking medical assistance in Somalia. The prevalence of HBV and HCV infections among dentistry patients is currently unknown. This study examined the seroprevalence of hepatitis B and C infections among patients, who visited dental care facilities in Mogadishu, Somalia.

## Method

### Study design and population

This study was conducted at Al Furaat Dental Clinic, one of the largest and most frequently visited private dental facilities in Mogadishu. The clinic receives patients from various districts of the city, providing a broad representation of the urban population seeking dental care. The study population included all patients who attended the dental clinic during the study period from February 2023 to March 2024.

### Sample size and sampling technique

The sample size for this study was calculated using Cochran’s formula for estimating a single population proportion. As there were no previous studies on the seroprevalence of Hepatitis B and C specifically within the dental patient population in Somalia, the estimated proportion was based on the most relevant available data. The seroprevalence of 18.9% for the general Somali population reported by Kadle et al. was used as the estimated proportion [21, 22]. The calculation was based on a 95% confidence level, a 5% margin of error, and a 5% non-response rate. This resulted in a minimum required sample size of 247 participants. A total of 410 participants were ultimately enrolled and included in the final analysis, exceeding the calculated minimum sample size, which provided the study with increased precision.

The formula used is:

$$n_0=\left(Z^2\times p\times q\right)/e^2$$

Where:

- *n*_*0*_​: Required sample size.
- *Z*: Z-value corresponding to the desired confidence level.
- *p*: Estimated proportion of the population with the attribute of interest.
- *q*: Proportion of the population without the attribute (1 − p).
- e: Desired margin of error.

A systematic random sampling technique was used to select the study participants [23]. Every third patient attending the dental clinic was invited to participate in the study from the total patient population who attended the clinic during the study period. The sampling continued until the final sample size of 410 participants was achieved, which exceeded the calculated minimum sample size of 247.

This study was approved by Jamhuriya University Research Ethics Committee (JUREC) under reference number JUREC0099/FMHS00313/052023.

### Data collection

Data was collected through face-to-face interviews using a structured questionnaire. Trained interviewers collected data on age, sex, marital status, education, vaccination status, transfusion history, surgical/dental procedures, frequency of dental visits, and chronic conditions. Participation was voluntary, and written informed consent was obtained before the interview and phlebotomy. Blood samples were also collected from each participant for serological testing of Hepatitis B surface antigen (HBsAg) and Hepatitis C antibody (anti-HCV). Before sample collection, informed consent was obtained from all participants. For minors (under 16 years of age), consent was obtained from their parents or guardians.

### Laboratory analysis

Venous blood samples were collected using plain tubes from each participant by a trained phlebotomist at the laboratory of Al Furaat Dental Clinic. The blood was then allowed to clot at room temperature for 30 min before being centrifuged at 3,000 revolutions per minute (rpm) for 10 min to obtain the serum. Serum specimens were then stored in a refrigerator at 4 °C until laboratory analysis was performed. Blood serum specimens were examined, with tests performed and interpreted strictly according to the manufacturers’ instructions. Commercially available rapid test strips and cassettes from InTec PRODUCTS, INC. in Xiamen, Fujian, P.R. China were used for HBsAg detection and anti-HCV detection [24, 25].

### Confirmation test using chemiluminescent immunoassay (CLIA)

Blood serum specimens that tested positive on the rapid test were analyzed using the MAGLUMI^®^ HBsAg chemiluminescence immunoassay (CLIA) kit from Snibe (Shenzhen, China) The assay quantitatively detects hepatitis B surface antigen (HBsAg) in human serum and plasma samples as an aid in diagnosing hepatitis B virus (HBV) infection. The test utilizes a sandwich chemiluminescence immunoassay principle. Samples are incubated with magnetic microbeads coated with monoclonal anti-HBs antibodies, forming antigen-antibody complexes. An ABEI-labeled polyclonal anti-HBs antibody is then added to form a sandwich immune complex. After magnetic separation and washing, a chemiluminescent reaction is initiated, and the emitted light signals are measured in relative light units (RLUs) by a photomultiplier. The RLUs correlate proportionally to the HBsAg concentration in the sample.

The assay was performed using a sample volume of 50 µL, and all reagents and calibrators were supplied ready-to-use. Samples and reagents were handled following the manufacturer’s biosafety and quality control protocols.

### Data analysis

Data was entered, and a thorough data cleaning process was performed to check for errors and handle any missing values. This ensured the integrity and accuracy of the dataset before any statistical analysis was conducted and analyzed using SPSS version 27.0. Descriptive statistics, such as frequencies and percentages, were calculated. Chi-square or Fisher’s exact tests assessed the association between seropositivity, demographic data, and medical history. p-value of less than 0.05 was considered statistically significant.

## Results

The study included 410 patients who visited Al Furaat Dental Clinic in Mogadishu, Somalia, from February 2023 to March 2024. The seroprevalence of HBV was 2.0% (95% CI: 0.9% to 3.6%), with no cases of HCV reported.

In terms of gender, female participants were higher (73.7%) than male participants (26.3%). The highest participation age group was 20–29 years old (41.2%). In addition, most individuals who visited dental care had a non-formal education (45.9%) (Table 1).

**Table 1:** The demographic data related to seroprevalence of HBV infection

Demographic data	No (%)	HBV positive No (%)	HBV negative No (%)	*P* value
Gender				1.000*
Male	108 (26.3)	2 (1.9)	106 (98.1)	
Female	302 (73.7)	6 (2.0)	296 (98.0)	
Age group				0.905*
10–19 years old	74 (18.0)	1 (1.4)	73 (98.6)	
20–29 years old	169 (41.2)	4 (2.4)	165 (97.6)	
30–39 years old	66 (16.1)	2 (3.0)	64 (97.0)	
40–49 years old	28 (6.8)	0 (0.0)	28 (100.0)	
50–59 years old	27 (6.6)	0 (0.0)	27 (100.0)	
60–69 years old	27 (6.6)	1 (3.7)	26 (96.3)	
70 + years old	19 (4.6)	0 (0.0)	19 (100.0)	
Marital status				1.000*
Single	242 (59.0)	5 (2.1)	237 (97.9)	
Married	137 (33.4)	3 (2.2)	134 (97.8)	
Divorced	17 (4.1)	0 (0.0)	17 (100.0)	
Widowed	14 (3.4)	0 (0.0)	14 (100.0)	
Educational Level				0.219*
Non-formal education	188 (45.9)	6 (3.2)	182 (96.8)	
Primary	115 (28.0)	0 (0.0)	115 (100.0)	
Secondary	72 (17.6)	1 (1.4)	71 (98.6)	
University/college	31 (7.6)	1 (3.2)	30 (96.8)	
Postgraduate	4 (1.0)	0 (0.0)	4 (100.0)	

*Fisher’s exact test was used due to an expected cell count of less than five

According to the relationship between the demographic data and the seroprevalence of HBV infection in this study, the prevalence of HBV in females (2.0%) was higher than in males (1.9%). While the highest prevalence of HBV was observed in the 60–69 years old age group. Regarding marital status, single individuals had the highest HBV seroprevalence (2.1%). The highest prevalence by educational level (3.2%) was observed among participants with non-formal education (Table 1).

Based on the medical history, 10.5% of individuals received blood transfusions, while only 3.2% had received a hepatitis B vaccination. The highest seroprevalence of HBV was reported among those who had received a blood transfusion (4.7%), compared to those who had not (1.6%). Notably, none of the individuals who tested positive for HBV had been vaccinated previously (Table 2). A prevalence of 2.6% was observed among participants without a history of surgery or dental interventions (such as tooth extraction or root canal treatment). The prevalence among those with no prior dental visits was 2.1%. Among participants without pre-existing chronic medical conditions, the prevalence was 1.8% (Table 2).

**Table 2:** The medical history related to seroprevalence of HBV infection

Medical history	No (%)	HBV positive No (%)	HBV negative No (%)	*P* value
Have you ever received a blood transfusion?				0.201*
yes	43 (10.5)	2 (4.7)	41 (95.3)	
No	367 (89.5)	6 (1.6)	361 (98.4)	
Have you ever been vaccinated for hepatitis B?				0.208*
Yes	13 (3.2)	0 (0.0)	13 (100.0)	
No	397 (96.8)	8 (2.0)	389 (98.0)	
Have you ever had surgery or other invasive dental procedures (e.g., tooth extraction, root canal)?				1.000*
Yes	101 (24.6)	0 (0.0)	101 (100.0)	
No	309 (75.4)	8 (2.6)	301 (97.4)	
How often do you visit the dentist?				1.000*
Never	387 (94.4)	8 (2.1)	379 (97.9)	
Less than once a year	10 (2.4)	0 (0.0)	10 (100.0)	
Once a year	3 (0.7)	0 (0.0)	3 (100.0)	
Twice a year	6 (1.5)	0 (0.0)	6 (100.0)	
More than twice a year	4 (1.0)	0 (0.0)	4 (100.0)	
Do you currently or have you previously had any chronic medical conditions (e.g., diabetes, liver disease)?				
Yes	27 (6.6)	1 (3.7)	26 (96.3)	0.423*
No	383 (93.4)	7 (1.8)	376 (98.2)	

*Fisher’s exact test was used due to an expected cell count of less than five

It is important to note that no statistically significant relationship was found between the demographic data or medical conditions and HBV seroprevalence (Tables 1 and 2).

## Discussion

Hepatitis B virus and HCV are infections of global concern [26]. Transmission can occur through needle pricks from infected patients, contaminated syringes, and inadvertent injections during surgical, dental, or other invasive procedures [27, 28]. This is the first study to investigate the seroprevalence of HBV and HCV among patients attending dental clinics in Mogadishu, Somalia. Data were collected from Al Furaat Dental Care. Owing to frequent occupational exposure to blood and saliva, dental healthcare providers are considered to be at heightened risk for hepatitis virus infections [28–30].

The current study reported a 2.0% seroprevalence of HBV infection and no cases of HCV infection. A similar study conducted in Peshawar, Pakistan, reported the seroprevalence of HBV rates of 2.1% [31]. Al-Amad also found the seroprevalence of HBV at 2.2% [16], which aligns with our results. In Africa, a study by Adewole et al. in Nigeria found that the overall HBV seroprevalence among dental patients was reported at 9.4% [32]. These findings suggest that dental practitioners encounter a significant risk of HBV infection among dental patients.

The seroprevalence of 2.0% in dental patients was low compared to 18.9% in a systematic review targeting the Somali general population [22], 3.6% among Somali immigrants in Minnesota, USA [33], and 9.7% among blood donors [34].

In the present study, HBV prevalence at 3.7% was highest among dental patients aged 60–69. Although Somali studies have not reported HBV prevalence by detailed age category specifically within dental patients, existing evidence shows HBV infection is consistently more common in adults compared to children and younger populations [34–36]. Our findings suggest that older adults are at higher risk of HBV infection.

The results of our study indicate that single individuals showed the highest HBV prevalence at 2.1%, while those with non-formal education had a prevalence of 3.2%. This aligns with findings from a study by Mohamud et al. in Somalia, which reported a notably high HBV prevalence of 60.4% among single individuals, emphasizing the significant risk associated with marital status [34]. Similarly, Hassan et al. found that among pregnant women in Mogadishu, 46.1% had no formal education, with an HBV prevalence of 14.8% in this group [37]. These findings highlight the critical role that social determinants, including marital status and educational attainment, play in influencing HBV infection risk. However, it is important to note that the associations were not statistically significant (*p* > 0.05).

Furthermore, in the current study, only 3.2% reported receiving a hepatitis B vaccination, which is a low rate. In comparison, studies conducted in Somalia reported HBV vaccination rates of 2.8%, 15%, 16.0%, and 33.4% [34, 38–40]. The lack of a statistically significant association between vaccination and HBV prevalence highlights a significant gap in immunization efforts. The hepatitis B vaccine serves as a crucial preventive measure, contributing to a significant decrease in the global spread of HBV.

Additionally, our study showed that 10.5% had a history of blood transfusions, which indicates a potential risk factor for hepatitis transmission. Medical facilities in Somalia face significant demand for blood transfusions alongside a high incidence of HBV infection, due to different factors [34, 36, 41, 42].

Finally, the present study revealed that 24.6% had undergone surgery or invasive dental procedures (e.g., tooth extraction, root canal), and no cases of HBV were reported, which suggests that the prevalence of these specific risk factors was relatively low. In comparison, a study conducted in Al Farsha, Saudi Arabia, reported that 24.6% patients had undergone surgical operations, and 29.9% of patients reported a history of tooth extraction [43]. Another study conducted in Ethiopia reported that 11.3% of participants had undergone surgical operations and 27% % reported a history of tooth extraction [44]. This disparity highlights the necessity for infection control protocols in dental clinics, especially in regions with poor vaccination rates, to reduce the risk of hepatitis transmission among patients. No statistically significant associations were observed between HBV prevalence and blood transfusion or surgical history (*p* > 0.05).

The current study has numerous limitations, including a limited sample size and the collection of samples only from one dental clinic. The lack of polymerase chain reaction (PCR) testing restricted the capacity to verify current infections. Furthermore, the cross-sectional design of the study prevents the establishment of cause-and-effect relationships. The reliance on self-reported data using a questionnaire introduces the possibility of recall and social desirability bias. Additionally, the small number of positive cases limited the ability to find statistically significant associations between seroprevalence and various demographic or medical history factors. Further studies need to include collecting data from many dental clinics to strengthen the validity of the results.

## Conclusion

This study highlights the seroprevalence of HBV and HCV among dental care patients in Mogadishu, Somalia, demonstrating a low HBV prevalence of 2.0% and no cases of HCV. The demographic data in this study showed a high prevalence among the age group of 60–69 years old and single individuals with non-formal education. Indicate that the result was not statistically significant. These results highlight the importance of targeted public health initiatives to enhance awareness and screening for HBV infection, as well as the importance of tailored educational interventions. Furthermore, this study represents a preliminary effort to comprehend HBV infection in Mogadishu, Somalia, highlighting the necessity of continuous surveillance and preventive strategies to reduce transmission risk and enhance health outcomes among dental patients in Somalia.

## Data Availability

Data are supplied in the manuscript.

## Abbreviations

HBV: Hepatitis B virus
HCV: Hepatitis C virus
HBsAg: Hepatitis B surface antigen Hepatitis B surface antigen
anti-HCV: Hepatitis C antibody
WHO: World Health Organization
CLIA: Chemiluminescent immunoassay
PCR: Polymerase chain reaction
